# Sources of variation in multicenter rectal MRI data and their effect on radiomics feature reproducibility

**DOI:** 10.1007/s00330-021-08251-8

**Published:** 2021-10-16

**Authors:** Niels W. Schurink, Simon R. van Kranen, Sander Roberti, Joost J. M. van Griethuysen, Nino Bogveradze, Francesca Castagnoli, Najim el Khababi, Frans C. H. Bakers, Shira H. de Bie, Gerlof P. T. Bosma, Vincent C. Cappendijk, Remy W. F. Geenen, Peter A. Neijenhuis, Gerald M. Peterson, Cornelis J. Veeken, Roy F. A. Vliegen, Regina G. H. Beets-Tan, Doenja M. J. Lambregts

**Affiliations:** 1 Department of Radiology, The Netherlands Cancer Institute, POB 90203, 1006 BE Amsterdam, The Netherlands; 2 GROW School for Oncology & Developmental Biology, University of Maastricht, Maastricht, The Netherlands; 3 Department of Radiation Oncology, The Netherlands Cancer Institute, Amsterdam, The Netherlands; 4 Department of Epidemiology and Biostatistics, The Netherlands Cancer Institute, Amsterdam, The Netherlands; 5 Department of Radiology, Acad. F. Todua Medical Center, Research Institute of Clinical Medicine, Tbilisi, Georgia; 6 Department of Radiology, Maastricht University Medical Centre, Maastricht, The Netherlands; 7 Department of Radiology, Deventer Ziekenhuis, Deventer, The Netherlands; 8 Department of Interventional Radiology, Elisabeth Tweesteden Hospital, Tilburg, The Netherlands; 9 Department of Radiology, Jeroen Bosch Hospital, ’s-Hertogenbosch, The Netherlands; 10 Department of Radiology, Northwest Clinics, Alkmaar, The Netherlands; 11 Department of Surgery, Alrijne Hospital, Leiderdorp, The Netherlands; 12 Department of Radiology, Spaarne Gasthuis, Haarlem, The Netherlands; 13 Department of Radiology, IJsselland Hospital, Capelle Aan Den IJssel, The Netherlands; 14 Department of Radiology, Zuyderland Medical Center, Heerlen, The Netherlands

**Keywords:** Multicenter study, Rectal neoplasms, Reproducibility of results, Magnetic resonance imaging, Image processing, Computer-assisted

## Abstract

**Objectives:**

To investigate sources of variation in a multicenter rectal cancer MRI dataset focusing on hardware and image acquisition, segmentation methodology, and radiomics feature extraction software.

**Methods:**

T2W and DWI/ADC MRIs from 649 rectal cancer patients were retrospectively acquired in 9 centers. Fifty-two imaging features (14 first-order/6 shape/32 higher-order) were extracted from each scan using whole-volume (expert/non-expert) and single-slice segmentations using two different software packages (PyRadiomics/CapTk). Influence of hardware, acquisition, and patient-intrinsic factors (age/gender/cTN-stage) on ADC was assessed using linear regression. Feature reproducibility was assessed between segmentation methods and software packages using the intraclass correlation coefficient.

**Results:**

Image features differed significantly (*p* < 0.001) between centers with more substantial variations in ADC compared to T2W-MRI. In total, 64.3% of the variation in mean ADC was explained by differences in hardware and acquisition, compared to 0.4% by patient-intrinsic factors. Feature reproducibility between expert and non-expert segmentations was good to excellent (median ICC 0.89–0.90). Reproducibility for single-slice versus whole-volume segmentations was substantially poorer (median ICC 0.40–0.58). Between software packages, reproducibility was good to excellent (median ICC 0.99) for most features (first-order/shape/GLCM/GLRLM) but poor for higher-order (GLSZM/NGTDM) features (median ICC 0.00–0.41).

**Conclusions:**

Significant variations are present in multicenter MRI data, particularly related to differences in hardware and acquisition, which will likely negatively influence subsequent analysis if not corrected for. Segmentation variations had a minor impact when using whole volume segmentations. Between software packages, higher-order features were less reproducible and caution is warranted when implementing these in prediction models.

**Key Points:**

*• Features derived from T2W-MRI and in particular ADC differ significantly between centers when performing multicenter data analysis.*

*• Variations in ADC are mainly (> 60%) caused by hardware and image acquisition differences and less so (< 1%) by patient- or tumor-intrinsic variations.*

*• Features derived using different image segmentations (expert/non-expert) were reproducible, provided that whole-volume segmentations were used. When using different feature extraction software packages with similar settings, higher-order features were less reproducible.*

**Supplementary Information:**

The online version contains supplementary material available at 10.1007/s00330-021-08251-8.

## Introduction

Over the past decade, more than 100 papers have been published on the use of MR imaging biomarkers to predict various clinical outcomes in rectal cancer such as treatment response and survival [1–3]. Imaging biomarkers range from relatively simple measures (tumor size and volume) [1, 2] to “functional” measures derived from imaging sequences such as diffusion-weighted imaging (DWI) and dynamic contrast-enhanced MRI[4]. More recently, the focus of research has shifted towards more advanced post-processing techniques such as radiomics used to extract large numbers of quantitative features to construct a radiological phenotype of the studied lesion [2, 5]. Common radiomics features include “first-order” histogram features (e.g., mean, standard deviation), shape features (e.g., volume, sphericity), and more complex higher-order texture features (e.g., gray-level co-occurrence matrix features) that describe patterns within the image.

While imaging biomarker studies have shown promising results to predict oncologic outcomes, several authors have voiced concern about the poor reproducibility and repeatability of these studies [6–8], related to small/underpowered single-center study designs, lack of independent model validation, and poor reproducibility of imaging features [6–8]. Important factors affecting reproducibility are data variations introduced by differences in acquisition, post-processing, and statistical analysis [9]. This is especially relevant for multicenter studies where data is generated using different hardware, software, and acquisition protocols, and where data is often evaluated by different readers. These variations are often referred to as “center effects” [10] and defined as “non-biological systematic differences between measurements of different batches of experiments” [11] that can negatively affect the performance of multicenter models [12].

Studies investigating sources of variation in imaging data have so far mainly focused on CT and PET and only one of 35 studies in a systematic review on radiomics feature reproducibility focused on MRI [9]. Some recent studies have explored variations in quantitative MRI analysis, though mainly in phantoms [13–16] or small (< 48 patients) single-center [13, 17, 18] or bi-institutional [19] patient cohorts. The current study aimed to add to these previous data by analyzing a large representative sample of rectal MRIs acquired at multiple institutions in the Netherlands to gain insight into how variations in “real life” clinical MRI data can affect radiomics studies. In specific, the goal was to investigate sources of variation focusing on hardware, image acquisition, and effects of post-processing related to segmentation methodology and feature extraction software.

## Materials and methods

### Patients

**Fig. 1:**
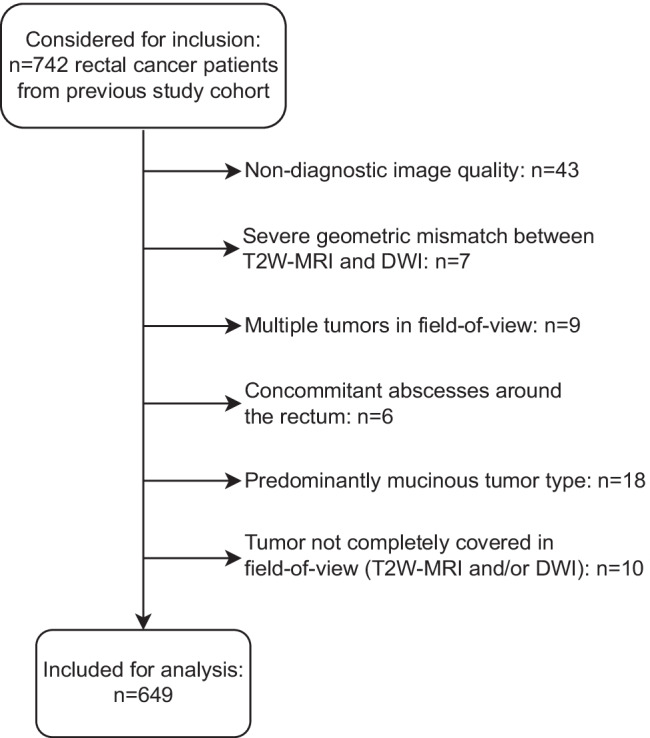
In- and exclusion flowchart

**Fig. 2:**
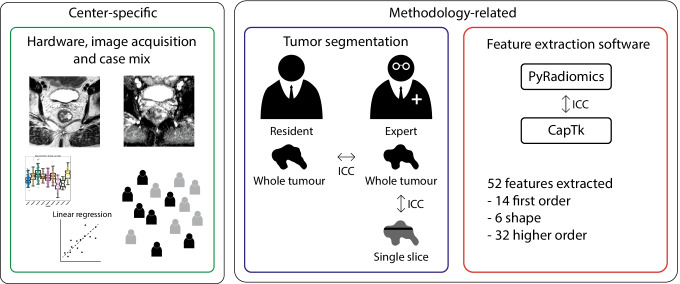
Study overview. Two types of data variation between centers were analyzed: center-specific variations (related to hardware and image acquisition protocols, and case-mix) and methodology-related sources of variation (related to segmentation and feature extraction methodology). ICC, intra-class correlation coefficient

For this retrospective study, we analyzed a dataset of rectal MRI scans (scanned between 2012 and 2017) previously collected as part of an ongoing IRB-approved retrospective multicenter study on prediction of response to neoadjuvant treatment, including patients from nine different centers in the Netherlands (1 tertiary oncologic referral center, 1 academic and 7 non-academic centers). Inclusion criteria for this previous study were (1) biopsy-proven rectal adenocarcinoma, (2) neoadjuvant treatment (chemoradiotherapy or 5 × 5 Gy radiotherapy with a long waiting interval) followed by surgery or watch-and-wait (W&W), (3) availability of baseline staging MRI (including T2W-MRI and DWI), and (4) availability of clinical outcome to establish response. From this initial cohort of 742 patients, 93 were excluded for reasons detailed in the in-/exclusion flowchart in Fig. 1, leaving a total study population of 649 patients. The overall study methodology is illustrated in Fig. 2.

### Imaging and image processing steps

All images were acquired according to routine practice in the participating centers using various vendors and acquisition protocols. The transverse T2W-MRI and apparent diffusion coefficient (ADC) maps were selected for analysis, as these were most commonly reported in previous rectal cancer image biomarker studies [2]. ADC maps were calculated from the DWI-series with a mono-exponential fit of the signal intensity using all available *b*-values (varying from 2 to 7 *b*-values per sequence; *b*-values ranging between b0 and b2000). Negative ADC values (< 0) or ADC values larger than 3 standard deviations from the mean (> mean + 3SD) were marked invalid. As T2W pixel values are represented on an arbitrary scale, these images were normalized to mean = 0 and standard deviation = 100. All images were then resampled to a common pixel spacing of 2 × 2 × 2 mm.

To explore the effects of segmentation methodology, three types of tumor segmentations were generated using 3D-slicer (version-4.10.2). Segmentations were generated on high *b*-value DWI, using the T2W-MRI as an anatomical reference, and then copied to the T2W-MRI and ADC maps. First, a non-expert reader (resident-level with no specific expertise in reading rectal MRI) segmented the rectal tumors by applying the “level-tracing” algorithm on DWI and manually adjusting it to exclude obvious artefacts or non-tumor tissues (e.g., adjacent organs or lymph nodes). Second, a board-certified radiologist (with > 10 years of experience in rectal MRI) manually revised these segmentations, taking care to precisely delineate the tumor boundaries slice-by-slice. Third, a single-slice segmentation was derived from this expert segmentation including the axial slice with the largest tumor surface area. These three segmentations will be further referred to as follows: (1) non-expert, (2) expert, and (3) single-slice segmentation.

Imaging features were extracted using PyRadiomics (version-v3.0). To explore the effects of feature extraction methodology, features (for the whole-volume expert-segmentations) were additionally extracted with similar software settings using a different open-source software package, CapTk (version-1.8.1). Only features defined in both software packages were extracted, including 52 features in total: 14 first-order, 6 shape, and 32 higher-order (7 gray-level co-occurrence matrix (GLCM), 16 gray-level size zone matrix (GLSZM), 4 gray-level run-length matrix (GLRLM), and 5 neighboring gray-tone difference matrix (NGTDM)).

## Analysis of sources of variation

### Center variations (case-mix, hardware, and image acquisition)

To investigate potential effects of “case-mix” differences, baseline patient characteristics were compared between centers using the Kruskal–Wallis test for age, T-stage, and N-stage, and chi-squared test for sex.

As a first exploratory step, we derived 6 basic imaging features (minimum, maximum, mean, standard deviation, entropy, and tumor volume) for each patient for both the T2W-MRI and ADC maps. The distribution of these features within our cohort was then visualized for each center separately using notched boxplots. To test whether the medians of the derived features were significantly different between patients from different centers we used the Kruskal–Wallis; to identify which specific centers have different feature distributions, a post hoc pairwise Mann–Whitney U-test was performed with Bonferroni correction to account for multiple testing. Supplementary Materials 1 describes a sub-analysis exploring whether differences between centers can be harmonized retrospectively by adjusting the b-values for ADC calculation or by performing data normalization using reference organs or z-transformation.

Using multivariable linear regression, we further explored the effects of variations in hardware (vendor/scanner model, field strength) and acquisition parameters (slice thickness, acquired in-plane resolution, repetition time, echo time, number of signal averages, maximum *b*-value, number of *b*-values, signal-to-noise ratio) on ADC and compared these to various patient-intrinsic (baseline and clinical outcome) parameters previously reported to be correlated with ADC (including sex, age, cT-stage, cN-stage, response to chemoradiotherapy and tumor volume [1, 2, 20]). “Center” (i.e., hospital) was investigated as a final parameter to account for unknown variations not covered by the other variables (e.g., patient preparation and undocumented acquisition parameters). Analyses were performed using R version-3.6.1, and *p* values < 0.05 were considered statistically significant. Further details on the regression analysis are provided in Supplementary Materials 2.

### Image segmentation

Imaging features were compared between the expert, non-expert, and single-slice segmentations using the two-way absolute agreement intra-class correlation coefficient (ICC), with ICC < 0.50 indicating poor agreement, 0.50 ≤ ICC < 0.75 moderate agreement, 0.75 ≤ ICC < 0.90 good agreement, and ICC > 0.90 excellent agreement [21].

### Feature extraction software

Imaging features derived with PyRadiomics (using expert segmentations) were compared to those derived using CapTk using the two-way absolute agreement ICC and the same cut-offs for agreement detailed above [21].

## Results

### Sources of variation

#### Center variations (patient-mix, hardware, and image acquisition)

**Fig. 3:**
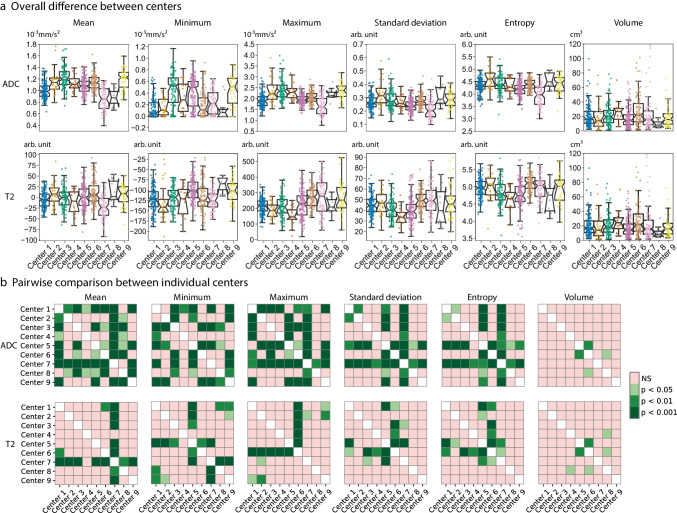
Center variations. **a** Visualization of the distribution of 6 basic (first-order + volume) imaging features within our study cohort, grouped by center. The imaging features were extracted from the rectal tumors on the ADC map (upper row) and T2W-MRI (bottom row), respectively. The boxplots show the distribution of the feature values for all patients within each center, with the notches in each box plot representing the 95% confidence intervals of the median feature value within a center. Kruskal–Wallis tests showed that for all features these median values were significantly different between the centers (*p* < 0.001). **b** Additional post hoc pairwise significance tests to explore which specific centers had significantly different feature values, with pink indicating no significant differences between centers and green indicating a significant difference (darker green corresponding to a higher level of significance). Bonferroni correction was used to account for multiple testing

Baseline characteristics of the 649 study patients (417 male, median age 65 years) are provided in Table 1. There were no significant differences in cT-stage, age, and sex distribution between the nine centers (*p* = 0.11–0.69), except for cN-stage that was significantly higher in one center (*p* < 0.001). An overview of the main variations in hardware and acquisition protocols is provided in Table 2. The distribution of basic first-order feature values per center and post hoc analyses illustrating differences between individual centers are depicted in Fig. 3. All tested features differed significantly between centers (Kruskal–Wallis *p* < 0.001) on both T2W-MRI and ADC. Pairwise comparisons between individual centers revealed that mainly ADC mean, minimum and maximum showed significant differences between the majority of the centers, while for T2W-MRI features, and ADC standard deviation and entropy, differences were limited to 2–4 individual centers. Data variations between centers did not improve after b-value harmonization and remained significant after applying different retrospective normalization methods, though normalization using inguinal lymph nodes as a reference organ did have a positive effect in reducing data variations as outlined in Supplementary Materials 1. Tumor volumes were mostly comparable between centers and only differed significantly between a minority of individual centers.

**Table 1:** Baseline patient characteristics and variations between centers

		Total	Center 1	Center 2	Center 3	Center 4	Center 5	Center 6	Center 7	Center 8	Center 9	*p* value
TOTAL, n (%)		*n* = 649 (100%)	*n* = 133 (20.5%)	*n* = 27 (4.2%)	*n* = 99 (15.3%)	*n* = 21 (3.2%)	*n* = 137 (21.1%)	*n* = 97 (14.9%)	*n* = 81 (12.5%)	*n* = 11 (1.7%)	*n* = 43 (6.6%)	
Sex, *n* (%)	Female	232 (35.7)	45 (33.8)	12 (44.4)	37 (37.4)	6 (28.6)	52 (38.0)	35 (36.1)	28 (34.6)	6 (54.5)	11 (25.6)	0.686*
	Male	417 (64.3)	88 (66.2)	15 (55.6)	62 (62.6)	15 (71.4)	85 (62.0)	62 (63.9)	53 (65.4)	5 (45.5)	32 (74.4)	
Age, median (range)		65 (26–88)	67 (31–87)	69 (41–88)	67 (26–86)	65 (46–77)	65 (39–86)	64 (35–83)	65 (44–82)	55 (32–79)	62 (44–80)	0.112**
cT, *n* (%)	2	40 (6.2)	7 (5.3)	1 (3.7)	7 (7.1)	1 (4.8)	11 (8.0)	4 (4.1)	6 (7.4)	0 (0.0)	3 (7.0)	0.228**
	3	523 (80.6)	101 (75.9)	21 (77.8)	75 (75.8)	16 (76.2)	112 (81.8)	83 (85.6)	65 (80.2)	11 (100.0)	39 (90.7)	
	4	86 (13.3)	25 (18.8)	5 (18.5)	17 (17.2)	4 (19.0)	14 (10.2)	10 (10.3)	10 (12.3)	0 (0.0)	1 (2.3)	
cN, *n* (%)	0	100 (15.4)	6 (4.5)	9 (33.3)	20 (20.2)	2 (9.5)	31 (22.6)	13 (13.4)	10 (12.3)	2 (18.2)	7 (16.3)	< 0.001**^, #^
	1	170 (26.2)	31 (23.3)	7 (25.9)	26 (26.3)	6 (28.6)	38 (27.7)	23 (23.7)	15 (18.5)	7 (63.6)	17 (39.5)	
	2	379 (58.4)	96 (72.2)	11 (40.7)	53 (53.5)	13 (61.9)	68 (49.6)	61 (62.9)	56 (69.1)	2 (18.2)	19 (44.2)	

^*^Calculated using the chi-squared test

^**^ Calculated using the Kruskal–Wallis test

^#^ Post hoc analysis using the pair-wise Mann–Whitney U test indicated that center 1 significantly differed from the other centers with respect to cN-stage (*p* < 0.001); no significant differences were detected between the remaining 8 centers

**Table 2:** Overview of main variations in hardware and acquisition protocols between the 9 participating centers

Hardware				
Total number of scanners				*n* = 26
Total number of scanner models				*n* = 13
Vendor type				
	Philips Healthcare (used in 6 centers)			*n* = 11 (incl. 4 different scanner models)
	Siemens Healthineers (used in 5 center)			*n* = 12 (incl. 7 different scanner models)
	GE Healthcare (used in 2 centers)			*n* = 3 (incl. 2 different scanner models)
Field strength				
	1.5 T		*n* = 19	
	3.0 T		*n* = 7	
Acquisition protocol				
Parameter		T2W-MRI median (range)		DWI median (range)
TR (ms)		4235 (866–16,738)		5475 (948–11,000)
TE (ms)		108 (60–250)		80 (37–117)
Flip angle (°)		150 (90–173)		90 (70–180)
NSA		2 (1–6)		5 (1–15)
Slice thickness (mm)		3 (3–5)		5 (2.7–8)
Pixel spacing (mm)		0.63 (0.29–1.48)		1.63 (0.63–3.52)
Field of view (mm)		200 (150–400)		320 (160–520)
Total number of *b*-values		N/A		3 (2–7)
Lowest *b*-value		N/A		0 (0–50)
Highest *b*-value		N/A		1000 (600–2000)

*NSA* number of signal averages, *T* Tesla, *TE* echo time, *TR* repetition time

The results of the regression models developed to investigate the influence of hardware, acquisition, and patient-intrinsic (baseline and clinical outcome) parameters on mean tumor ADC are reported in Table 3. Acquisition parameters had the strongest association with ADC and on their own were able to explain 64.3% of the variation in ADC present in the data. Patient-intrinsic parameters (e.g., age, gender, TN-stage, treatment response) had a negligible effect on ADC and were able to explain only 0.4% of the variation in ADC. The umbrella variable “Center” was able to explain 32.5% of the variation in ADC. When combining all factors in one model, the model explained 63.5% of the data variation, with acquisition and hardware parameters as the main predictors.

**Table 3:** Factors attributing to mean tumor ADC

Factors:	Proportion of variance in ADC predicted by these factors (LOOCV *R*^2^)
A. Hardware and acquisition parameters:	64.3%
Repetition time (TR)*	
Echo time (TE)*	
Flip angle	
Pixel Bandwidth	
In plane resolution*	
Slice thickness	
Number of signal averages (NSA)*	
Maximum *b*-value*	
Number of *b*-values*	
Signal to noise ratio (SNR)*	
Scanner model*	
Magnetic field strength	
B. Patient-intrinsic parameters	0.4%
Age	
Sex	
cT-stage (assessed on baseline MRI)	
cN-stage (assessed on baseline MRI)	
Response to chemoradiotherapy (complete versus incomplete response)	
Tumor volume	
C. Center	32.5%
Umbrella variable to account for any additional unknown variations between centers (e.g., patient preparation protocols, types of coils used, fat suppression techniques, etc.)	
Significant parameters: center	
All (A + B + C) combined	63.5%
Significant parameters: center, age, TR, TE, in-plane resolution, slice thickness, NSA, maximum *b*-values, number of *b-*values, scanner model, and SNR	

Further details of the regression analysis can be found in Supplementary Materials 2. The LOOCV *R*^2^ value is a leave-one-out cross-validated goodness-of-fit measure indicating the proportion of the variance in the dependent (i.e., ADC) variable that can be explained by the independent variables using a linear regression model

^*^Indicate the variables that were significant with a *p* value < 0.05 based on a likelihood ratio test. For continuous variables, all polynomial terms were tested jointly

#### Image segmentation

**Fig. 4:**
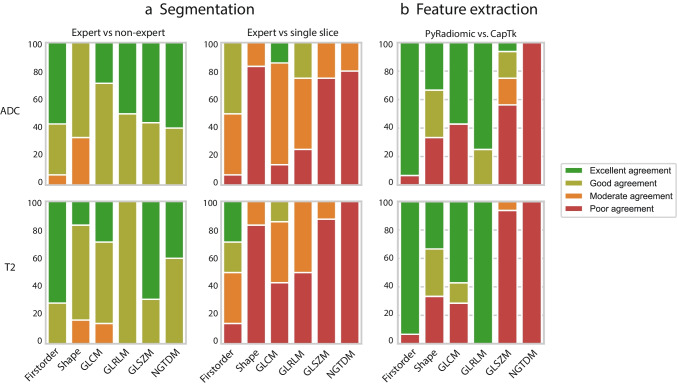
Effects of image segmentation and feature extraction methodology. Feature reproducibility for ADC (upper row) and T2W-MRI (bottom row) using different segmentation methods (**a**) and feature extraction packages (**b**). Each column corresponds to the percentage of features showing excellent (dark green, ICC > 0.90), good (green, 0.90 > ICC > 0. 75), moderate (orange, 0.75 > ICC > 0.5) or poor (red, ICC < 0.5) agreement. In total, 52 features were analyzed, including 14 first-order, 6 shape, 7 gray-level co-occurrence matrix (GLCM), 4 gray-level run-length matrix (GLRLM), 16 gray-level size zone matrix (GLSZM), and 5 neighboring gray-tone difference matrix (NGTDM) features

The results of the reproducibility analysis using different segmentation strategies are depicted in Fig. 4A. Reproducibility between expert and non-expert segmentations was good–excellent for the majority of features (first-order, shape, and higher-order) with ICC values ranging between 0.72 and 0.99 (median 0.90) for T2W-MRI and 0.53 and 0.99 (median 0.89) for ADC. Compared to the expert whole-volume segmentations, the extracted single-slice segmentations resulted in considerably lower reproducibility with an ICC of 0.00–0.94 (median 0.40) for T2W-MRI and ICC of 0.00–0.97 (median 0.58) for ADC, with poor results for shape, GLSZM, and NGTDM features.

#### Feature extraction software

The influence of feature extraction software is depicted in Fig. 4B. The majority of first-order, shape, GLCM, and GLRLM features showed good–excellent reproducibility with similar results for features derived from T2W-MRI or ADC with ICCs ranging between 0.00 and 1.00 (median 0.99) for both modalities. In contrast, the majority of GLSZM and NGTDM features were poorly reproducible with ICCs of 0.00–0.56 (median 0.00) for T2W-MRI and ICCs 0.01–0.99 (median 0.41) for ADC.

## Discussion

The results of this study show that variations in quantitative imaging (radiomics) in a large clinical multicenter dataset of rectal MRIs were more substantial for DWI/ADC than for T2W-MRI and mainly related to hardware and acquisition protocols (i.e., “center effects”). The effects of segmentation methodology and feature extraction software on feature variation were less significant, particularly for the more basic first-order and shape-related features that showed overall good reproducibility.

An exploratory analysis on the feature distribution of 6 basic imaging features (first-order + volume) showed significant variation between patient populations coming from different centers, with more significant differences for ADC than for T2W-MRI. Tumor volume was the most robust feature with the most comparable results between centers. This is in line with a previous report on the repeatability of MRI features in a small cohort (*n* = 48) of patients with brain glioblastoma, which showed that shape features (including volume) resulted in higher repeatability than features derived from T2W-MRI pixel intensities [18]. Similarly, shape features were found to have the highest repeatability and reproducibility on T2W-MRI of cervical cancer [22].

Since ADC data showed the largest variations, we developed a linear regression model to investigate in-depth which factors influence mean tumor ADC. The majority (> 60%) of the ADC variation could be predicted using only hardware and acquisition-related parameters while patient-intrinsic (clinical and tumor) features alone predicted only 0.4% of the variation in ADC. This suggests that—when building multicenter prediction models—any potential relation between clinical outcomes and ADC will likely be obscured when no correction is performed to account for acquisition and hardware differences. This is a very relevant issue when incorporating ADC into retrospective multicenter studies without protocol standardization. Even in controlled prospective study designs with harmonized acquisition protocols, variation in ADC can still be a limiting factor as coefficients of variation range as high as 4–37% depending on the measured organ [23, 24]. Differences in acquisition settings have previously also been shown to substantially reduce inter- and intra-scanner reproducibility of T2W-MRI radiomics features, with particularly poor results for higher-order features [13, 16].

Several methods have been suggested to correct for data variations between centers. A first option is to discard features that are poorly reproducible across centers, with the advantage of creating simpler models (though with the drawback of losing potentially valuable information) [12]. Two alternative options are normalization in the image domain (e.g., z-transform or within patient normalization using reference tissues) or feature domain (e.g., ComBat harmonization). These techniques have been shown to significantly improve T2W-MRI feature reproducibility [18] and to successfully correct for “batch-effects” (similar to “center-effects”) in genomic studies [12]. The latter approach has recently been adopted for radiomics with promising results [25]. In our exploratory analysis described in Supplementary Materials 1, data normalization using lymphoid tissue (benign inguinal lymph nodes) as a reference organ had a positive effect to reduce ADC data variations between centers, though differences remained statistically significant and the benefits of this approach will need to be further investigated in studies where features are tested against a clinical outcome. The fourth option is to use statistical models that specifically take center effects into account (e.g., random/mixed-effect models [10]). These various options all have their strengths and weaknesses and evidence-based guidelines on the preferred (combination of) methods to handle center effects in multicenter radiomics research are so far lacking and urgently needed.

Regarding image segmentation methodology, we found poor reproducibility for higher-order features (e.g., GLSZM and GLRLM) but overall good reproducibility for simpler features (e.g., first-order, GLCM) similar to previous reports [9, 26, 27]. The features derived from single-slice segmentations showed the poorest reproducibility, which is in line with previous single-center reports [28, 29], indicating that—though less cumbersome—single-slice methods are not recommendable. Interestingly, feature reproducibility was good–excellent between expert and non-expert readers, indicating that input from expert-radiologists is not necessarily required. This is in line with a previous report where a Radiomics model was trained to predict response to chemoradiotherapy in rectal cancer and achieved similar performance regardless of whether segmentations were performed by expert (AUC 0.67–0.83) or non-expert readers (AUC 0.69–0.79) [30]. This is reassuring given the tremendous workload associated with image segmentation, especially when analyzing large volumes of imaging data. Another potential solution to reduce this workload could be to use computer algorithms to (semi-)automatically generate tumor segmentations [31]. There have been some promising reports showing that computer algorithms may generate segmentations similar to manual tumor delineations [31, 32], provided that image quality is good [33].

When comparing feature reproducibility using different software packages, we found that the majority of first-order, shape, GLCM, and GLRLM features showed good to excellent reproducibility whereas the majority of higher-order features (GLSZM and NGTDM) were poorly reproducible. This is in line with earlier findings that higher-order features are generally less reproducible than first-order and shape features [9] which can probably be partly attributed to technical differences in the implementation of features and/or image processing by different software and computational algorithms. This underlines the importance of accurately reporting software versions, and preferably using packages with standardized feature definitions such as those defined by the image biomarker standardization initiative (IBSI). Considering the poor reproducibility of the higher-order features in our dataset, caution should be taken when incorporating more advanced features into clinical prediction models.

The main novelty of the current study lies in its multicenter aspect. Although previous studies have already identified acquisition parameters [13, 15, 16], segmentation [19], and post-processing methods [15, 18] as factors affecting feature reproducibility, these studies have so far mainly been performed on non-MRI data or in small patient cohorts or phantoms. The extent to which these effects influence feature reproducibility and may obscure correlations with common clinical outcomes in a representative “real life” clinical cohort of MRI data acquired at various institutions has not been previously reported. There were, however, some limitations to our study design in addition to its retrospective nature. As the data was acquired and anonymized to comply with privacy regulations, only basic acquisition information could be extracted from the DICOM headers. Other potential sources of variation, such as coil use, fat suppression, MRI software version, and patient preparation, therefore, remain underexposed. In addition, all segmentations were done on the high *b*-value DWI (and then copied to the other modalities). Although care was taken to take the anatomical information of T2W-MRI into account during the segmentations, ideally a separate segmentation would have been performed on T2W-MRI. This, however, was not feasible to accomplish within an acceptable timeframe. Finally, several data-processing choices (e.g., resampling voxel size, bin-width, gray-level discretization, and T2W-MRI normalization) were made which may have influenced the extracted features [34–36]. Although some of these steps may have introduced some bias in our analyses a more detailed analysis on the impact of these choices was beyond the scope of this paper.

In conclusion, this study has shown that significant variations between centers are present in multicenter rectal MRI data with more substantial variations in DWI/ADC compared to T2W-MRI, which are mainly related to hardware and image acquisition protocols (i.e. “center effects”). These effects need to be accounted for when analyzing multicenter MRI datasets to avoid overlooked potential correlations with the clinical outcome under investigation. Image segmentation has relatively minor effects on image quantification provided that whole-volume segmentations are performed. Expert segmentation input is not necessarily required to acquire stable features, which could shift the daunting task of image segmentation from expert-radiologists to less experienced readers or even (semi-)automatic software algorithms. Higher-order features were less reproducible between software packages and caution is therefore warranted when implementing these into clinical prediction models.

## Supplementary Information

Below is the link to the electronic supplementary material.

Supplementary file1 (DOCX 1153 KB)

## Abbreviations

GLCM: Gray-level co-occurrence matrix
GLRLM: Gray-level run-length matrix
GLSZM: Gray-level size zone matrix
Gy: Gray
ICC: Intra-class correlation coefficient
NGTDM: Neighboring gray-tone difference matrix
W&W: Watch-and-wait

## Declarations

### Guarantor

The scientific guarantor of this publication is Dr. Doenja MJ Lambregts.

### Statistics and biometry

One of the authors, Mr. Sander Roberti, has significant statistical expertise.

### Study subjects or cohorts overlap

Some (45/649) study subjects or cohorts have been previously reported in Lambregts (2018) Dis Colon Rectum.

### Methodology

• observational

• observational

• multicenter study
