# Supplementary material for: Sources of variation in multicenter rectal MRI data and their effect on radiomics feature reproducibility
Source: Eur Radiol. 2021 Oct 16;32(3):1506–16. doi: 10.1007/s00330-021-08251-8 (PMC8831294; doi:10.1007/s00330-021-08251-8)
Supplement: Supplementary file 1 — Supplementary file1 (DOCX 1153 KB) [file 330_2021_8251_MOESM1_ESM.docx]

# Supplementary Materials 1 – Data harmonization

Rationale and method:

In our exploratory analysis (**Figure 3**), significant differences were found in the median values of 6 basic imaging features between centers, which were most pronounced for features derived from ADC. With this additional analysis we aimed to investigate whether differences between centers can be harmonized using several simple normalization methods:

1. **B-value harmonization:**  in addition to the original ADC-maps, which were calculated including all available b-values as acquired in the respective participating centers, ADC-maps were re-calculated using only b0 (which was available in each center) and a high b-value as close as possible to b1000 (ranging from b600-b1000 in our dataset). The rationale is that the range and choice of b-values can affect the calculated ADC [1], and that using a similar number/range of b-values might improve the harmonization across centers.
2. **Normalization using a reference organ/tissue:** Previous studies have shown potential benefit for ADC normalization by calculating the ratio between tumour ADC features and those of a reference organ or tissue [2]. For this study we chose to perform standardized reference measurements in a non-pathologic inguinal lymph node (2a) and the urinary bladder (2b) as these reference sites were both available within the FOV of each of the studied patients in the dataset.
   1. **Lymph Node:** Reference measurements were performed by placing a single-slice region of interest (ROI) in a single morphologically non-suspicious node in the right or left inguinal region. Normalized ADC features were then calculated by dividing the tumour measurements by the mean pixel intensity of an inguinal lymph node
   2. **Urinary bladder:** The reference ADC was normalized by dividing the image by the mean pixel intensity of the bladder content.
3. **Z-transform:** A previous study showed that using z-score normalization improved feature reproducibility for T2-weighted FLAIR and T1-weighted post contrast images [3]. ADC was normalized by performing a z-transform, transforming the image to have mean=0 and standard deviation=1.

These methods were all compared to the original ADC calculations described in the main body of the manuscript, including all available b-values and without any retrospective normalization. As the range of image intensities changed as a result of normalization, different bin widths were used for different normalized images to ensure that the features were calculated using 20-130 bins as recommended in the PyRadiomics documentation. The bin width was 5e-5 for the original ADC calculations and method 1 (b-value harmonization), and 5e-2 for methods 2 and 3 (normalization using reference organ and z-transformation).

Statistical analysis:

In line with the methods described in the main manuscript, distributions of the ADC feature values acquired using the different methods described above were grouped by center and depicted using notched boxplots. Kruskal-Wallis test was then used to test whether the medians of the derived features were significantly different between patient populations coming from different centers. For each method a post-hoc analysis was performed using pairwise Mann-Whithney U-test with Bonferroni correction to account for multiple testing.

Results:

The distributions of the calculated features using the orginal (non-normalized) ADC maps and after applying the different normalization methods are depicted in **Supplementary Figure 1**. Kruskal-Wallis test was significant for all variables (p<0.001), regardless of which (if any) normalization method that was applied. Features that were normalized using an inguinal lymph node as a reference tissue did show substantially less variations between individual centers compared to the other methods, as is shown in the post-hoc analysis depicted in **Supplementary Figure 2**.

**
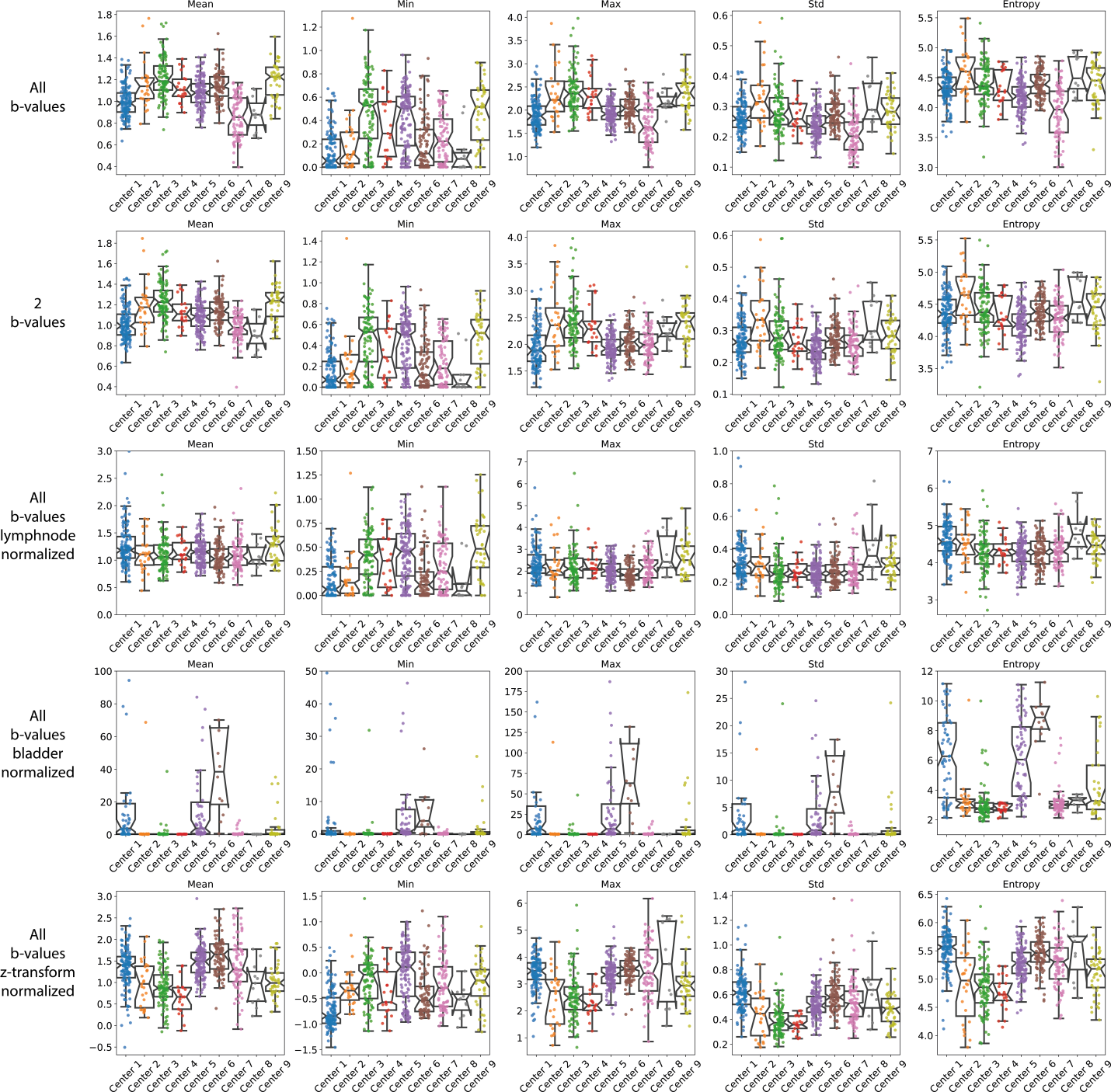
**

**Supplementary Figure 1:** Visualization of the distribution of 5 basic (first-order) imaging features within our study cohort, grouped by center. Features Rows (from top to bottom) represent features calculated from [1] the original ADC-maps, calculated including all available b-values, [2] ADC-maps calculated with 2 b-values, [3] ADC normalized using an inguinal lymph node as a reference organ, [4] ADC normalized using the urinary bladder as reference organ, and [5] ADC normalized by using a z-transformation (mean=0, standard deviation=1)

**
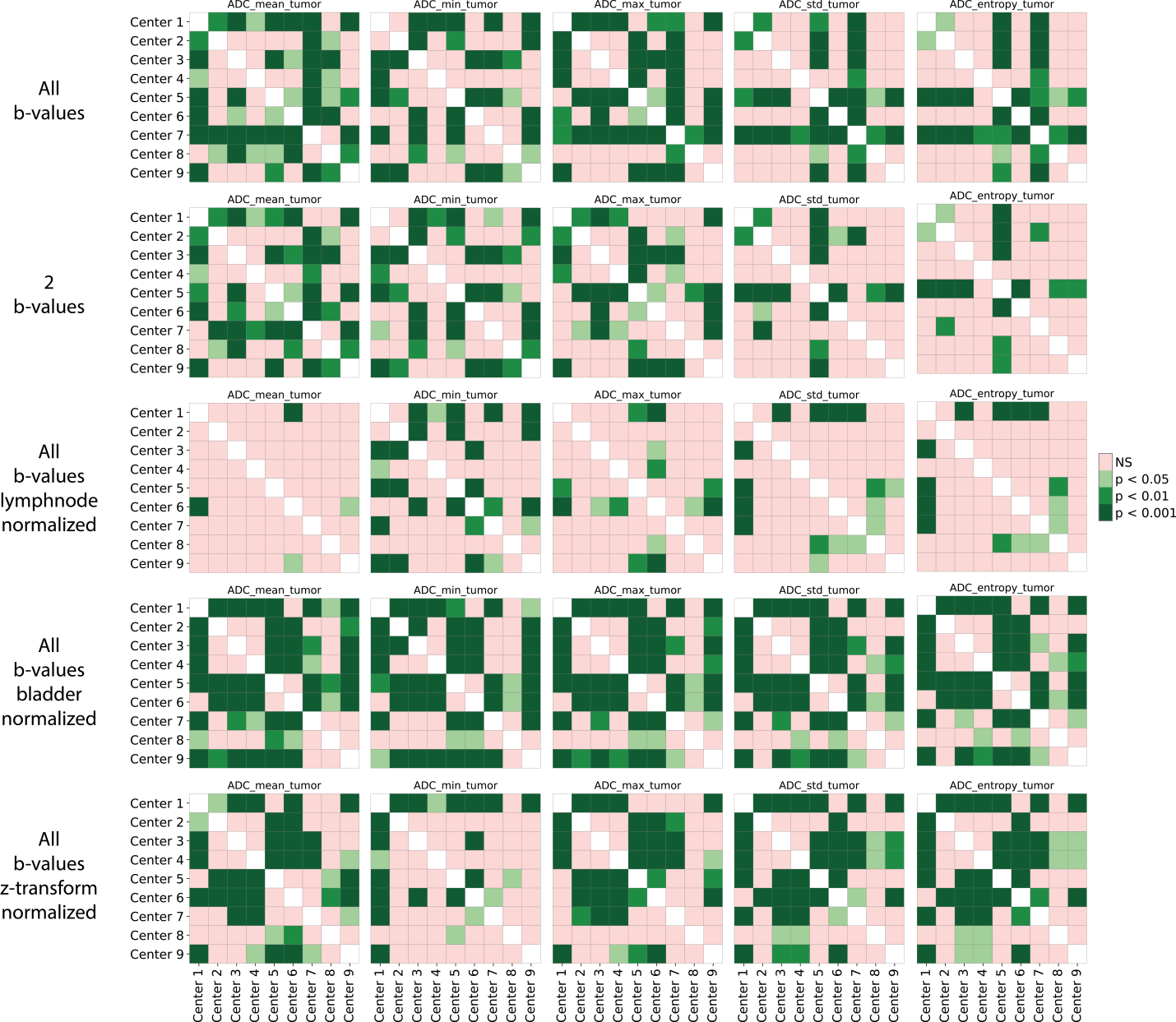
**

**Supplementary Figure 2:** Pairwise Mann-Whithney U significance test with Bonferroni correction, comparing the median feature values between different centers. The columns from left to right represent the mean, minimum, maximum, standard deviation and entropy derived from the tumor ADC. The ADC map was calculated and/or normalized according to different methods. There methods are depicted from top to bottom: ADC calculated with all b-values, ADC calculated with 2 b-values, ADC normalized using an inguinal lymph node as reference organ, ADC normalized using the bladder as reference organ, ADC normalized by using a z-transformation (mean=0, standard deviation=1)

**References**

1. Iima M, Partridge SC, Le Bihan D (2020) Six DWI questions you always wanted to know but were afraid to ask: clinical relevance for breast diffusion MRI. Eur Radiol 30:2561–2570. https://doi.org/10.1007/s00330-019-06648-0

2. Koc Z, Erbay G, Karadeli E (2017) Internal comparison standard for abdominal diffusion-weighted imaging. Acta radiol 58:1029–1036. https://doi.org/10.1177/0284185116681040

3. Hoebel K V., Patel JB, Beers AL, et al (2021) Radiomics Repeatability Pitfalls in a Scan-Rescan MRI Study of Glioblastoma. Radiol Artif Intell 3:e190199. https://doi.org/10.1148/ryai.2020190199

# Supplementary Materials 2

## Linear regression model to predict mean tumor ADC using hardware, image acquisition and patient intrinsic (clinical baseline and outcome) parameters

Rationale and method:

In our exploratory analysis (**Figure 3**), significant differences in the median values of 6 basic imaging features between centers were found which were most pronounced for features derived from ADC, in particular mean, minimum and maximum ADC. With this multivariable linear regression analysis we aimed to further investigate to what extent various factors might be able to explain these differences in ADC between centers. The mean tumor ADC was chosen as the dependent variable as it is an absolute quantifiable measure, as well as the ADC-parameter that has been most widely studied as a biomarker to predict a variety of clinical outcomes and tumor risk factors in rectal cancer [1-4]. From these previously reported clinical factors we selected cT-stage, cN-stage, tumor volume and response to chemoradiotherapy (complete versus incomplete) as patient/tumor-intrinsic input variables in this “reversed” regression model to predict ADC. Sex and age were added as additional clinical baseline parameters.

In addition we included several hardware (vendor/scanner model, field strength) and acquisition parameters (slice thickness, acquired in-plane resolution, repetition rime, echo time, number of signal averages, maximum b-value, number of b-values, signal-to-noise ratio), which were extracted from the image DICOM headers, except for the signal-to-noise ratio which was determined by dividing the average tumor ADC with the standard deviation of the background noise. The background noise standard deviation was estimated using the “estimate_sigma” function in the Diffusion Imaging in Python toolkit (DIPY; version 1.2.0) [5-6]. Finally, we included ‘Center’ as a categorical parameter to account for any systematic differences in ADC between centers related to factors not covered by the abovementioned clinical, hardware and acquisition parameters (such as patient preparation protocols, coils and coil elements used, additional acquisition parameters that could not be derived from the DICOM headers, etc.).

Statistical analysis and results:

Using the parameters groups defined above, 4 linear regression models were developed to predict ADC, based on:

1. Hardware and acquisition
2. Patient- and tumor- intrinsic (baseline & clinical outcome)
3. Center
4. All (A + B + C) combined

In these models cT-stage, cN-stage, field strength, scanner model, sex and center were treated as factor variables, with all other variables treated as continuous variables. The starting point was a linear model using all variables, for which visual inspection of residual plots indicated no problematic model misspecification. To improve model fit, polynomial terms of order ≥2 for each continuous variable were tested using likelihood ratio tests. If additional polynomial terms were significant these were added to the model. This was done sequentially, adding higher-order terms to the starting model until the next term was no longer significant. Model D was then obtained as the basic linear model with all significant polynomial terms added to it. Interactions between variables were not considered as theoretical support for a priori selection of interactions was limited, and there was a risk of overfitting when using an automatic model selection procedure such as stepwise selection. Model performance was assessed using the coefficient of determination R^2^ which is a goodness-of-fit measure indicating the proportion of the variance in the dependent variable (i.e. mean tumor ADC) explained by the linear regression model. To validate our results we performed leave-one-out cross-validation (LOOCV) for each model by excluding a single patient, fitting the model on the remaining patients (including forward selection of polynomial terms), and predicting the ADC for the left-out patient. The LOOCV R^2^ was then obtained as the squared Pearson correlation between the ADC predictions and the true ADC.

The performance of the tested models after LOOCV is reported in **Table 3**. Acquisition parameters had the strongest association with ADC and were able to explain 64.3% of the variation (LOOCV R^2^). Effects of patient-intrinsic baseline and clinical outcome parameters were negligible and on its own were able to explain 0.4% of the variation in ADC with age as the only significant predictor. The variable “center” on its own explained 32.5% of the variation. While the center variable was significant in the combined model containing all variables (model D) (p=2.82x10e^-6^), no benefit of the variable was observed in the R^2^ after cross-validation. This indicates a potential overfitting issue, however given that the LOOCV R^2^ is only slightly smaller for the full model (model D) than a model using a considerably smaller number of parameters (model A), the effect of overfitting is likely not problematic.

The model coefficients of the linear regression model based on all available variables are reported in **Supplementary Table 1** . Main predictive parameters were center, slice thickness, in plane resolution, repetition time, n^o^ of signal averages and maximum b-value.

| **Table 1:** Model coefficients | | | |
| --- | --- | --- | --- |
| **Parameter** | **Coefficient** | **P-value** |  |
| (Intercept) | 1.29E+00 |  |  |
| Center 2 | 6.56E-03 | 2.82E-06 | *** |
| Center 3 | 8.56E-02 |  |  |
| Center 4 | -5.06E-02 |  |  |
| Center 5 | -3.14E-02 |  |  |
| Center 6 | -3.75E-02 |  |  |
| Center 7 | -6.12E-02 |  |  |
| Center 8 | -2.83E-01 |  |  |
| Center 9 | 3.40E-02 |  |  |
| Male sex | 1.31E-02 | 1.20E-01 |  |
| Age (yr) | -2.24E-02 | 1.53E-01 | * |
| Age^2^ | 1.63E-04 |  |  |
| Age^3^ | 2.28E-06 |  |  |
| Age^4^ | -2.32E-08 |  |  |
| cT3 | -6.40E-03 | 8.39E-01 |  |
| cT4 | -1.17E-02 |  |  |
| cN1 | -2.61E-03 | 9.80E-01 |  |
| cN2 | -1.69E-03 | 8.94E-01 |  |
| Complete response to chemotherapy (pCR) | -7.52E-03 | 4.02E-01 |  |
| Slice thickness (mm) | 2.06E-02 | 3.03E-03 | ** |
| In plane resolution (mm) | 1.38E-01 | 1.12E-09 | *** |
| Repetition time (ms) | -2.27E-04 | 1.53E-03 | *** |
| Repetition time^2^ | 3.01E-08 |  |  |
| Repetition time^3^ | -1.22E-12 |  |  |
| Echo time (ms) | 1.70E-02 | 6.49E-03 | ** |
| Echo time^2^ | -9.44E-05 |  |  |
| Number of signal averages | -7.19E-02 | 6.80E-06 | *** |
| Number of signal averages^2^ | 9.92E-03 |  |  |
| Number of signal averages^3^ | -4.51E-04 |  |  |
| Field strength 3.0T | 2.53E-02 | 5.60E-01 |  |
| Maximum b-value | -1.37E-03 | 1.69E-23 | *** |
| Maximum b-value^2^ | 4.54E-07 |  |  |
| Number of b-values | 3.92E-01 | 9.37E-05 | *** |
| Number of b-values^2^ | -1.41E-01 |  |  |
| Number of b-values^3^ | 2.51E-02 |  |  |
| Number of b-values^4^ | -1.88E-03 |  |  |
| Scanner model Achieva dStream | -1.43E-03 | 2.94E-16 | *** |
| Scanner model Aera | -7.84E-02 |  |  |
| Scanner model Avanto | -4.10E-02 |  |  |
| Scanner model Avanto_fit | 1.68E-01 |  |  |
| Scanner model Espree | -9.67E-03 |  |  |
| Scanner model Ingenia | -3.42E-02 |  |  |
| Scanner model Intera | -8.79E-02 |  |  |
| Scanner model Optima MR450w | 2.19E-01 |  |  |
| Scanner model Signa HDxt | 1.05E-01 |  |  |
| Scanner model SymphonyTim | 1.41E-01 |  |  |
| Scanner model Verio | -1.25E-03 |  |  |
| Tumor volume (mm^3^) | -2.08E-07 | 2.88E-01 |  |
| SNR | 9.46E-01 | 7.80E-84 | *** |
| SNR^2^ | -1.19E+00 |  |  |
| SNR^3^ | 6.20E-01 |  |  |
| SNR^4^ | -1.55E-01 |  |  |
| SNR^5^ | 2.00E-02 |  |  |
| SNR^6^ | -1.29E-03 |  |  |
| SNR^7^ | 3.32E-05 |  |  |
| Flip angle (°) | 1.96E-04 | 2.00E-01 |  |
| Pixel bandwidth (Hz) | 1.91E-06 | 8.04E-01 |  |
| *** indicates p<0.001, ** indicates p<0.01, * indicates p<0.05  NB. p-value determined using likelihood ratio test. | | | |

**References**

1. Schurink NW, Lambregts DMJ, Beets-Tan RGH (2019) Diffusion-weighted imaging in rectal cancer: current applications and future perspectives. Br J Radiol 92:20180655
2. Joye I, Deroose CM, Vandecaveye V, Haustermans K (2014) The role of diffusion-weighted MRI and 18F-FDG PET/CT in the prediction of pathologic complete response after radiochemotherapy for rectal cancer: A systematic review. Radiother Oncol 113:158–165
3. Di Re AM, Sun Y, Sundaresan P, et al (2020) MRI radiomics in the prediction of therapeutic response to neoadjuvant therapy for locoregionally advanced rectal cancer: a systematic review. Expert Rev Anticancer Ther. <https://doi.org/10.1080/14737140.2021.1860762>
4. Attenberger UI, Pilz LR, Morelli JN, et al (2014) Multi-parametric MRI of rectal cancer - Do quantitative functional MR measurements correlate with radiologic and pathologic tumor stages? Eur J Radiol 83:1036–1043
5. Coupe P, Yger P, Prima S, et al (2008) An optimized blockwise nonlocal means denoising filter for 3-D magnetic resonance images. IEEE Trans Med Imaging 27:425–441
6. Garyfallidis E, Brett M, Amirbekian B, et al (2014) Dipy, a library for the analysis of diffusion MRI data. Front Neuroinform 8:1–17
